# Practical application of Octavius^®^‐4D: Characteristics and criticalities for IMRT and VMAT verification

**DOI:** 10.1002/acm2.12412

**Published:** 2018-07-16

**Authors:** Patrizia Urso, Rita Lorusso, Luca Marzoli, Daniela Corletto, Paolo Imperiale, Annalisa Pepe, Lorenzo Bianchi

**Affiliations:** ^1^ Department of Medical Physics A.S.S.T. Valle Olona Busto Arsizio Italy; ^2^ Present address: Department of Radiotherapy Clinica Luganese Moncucco SA Lugano Switzerland

**Keywords:** IMRT, Octavius^®^‐4D, quality assurance, VMAT, *γ*‐index

## Abstract

Octavius^®^‐4D is a very effective device in radiotherapy treatment quality assurance (QA), due to its simple set‐up and analysis package. However, even if it is widely used, its main characteristics and criticalities were only partially investigated. Taking start from its commissioning, the aim of this work was to study the main dependencies of the device response. The outcome dependence was studied comparing results by different delivery techniques [Intensity Modulated Radiation Therapy, IMRT (*n* = 29) and RapidArc, RA (*n* = 15)], anatomical regions [15 head/neck, 19 pelvis and 10 pancreas] and linear accelerators [DHX (*n* = 14) and Trilogy (*n* = 30)]. Moreover, the agreement dependency on the section of the phantom was assessed. Plan evaluations obtained by 2D, 3D, and volumetric *γ*‐index (both local and global) were also compared. Generally, high dose gradient resulted critically managed by the assembly, with a smoother effect in RA technique. Worse agreements emerged in the 2D *γ*‐index vs those of 3D and volumetric (*P* < 0.001), that were instead statistically comparable in global metric (*P* > 0.300). Volumetric plan evaluation was coherent with the average of passing rates on the 3 phantom axes (*r* ≥ 0.9), but transversal section provided best agreements vs sagittal and coronal ones (*P* < 0.050). The three studied districts furnished comparable results (*P* > 0.050) while the two LINACs provided different agreements (*P* < 0.005). The study pointed out that the phantom transversal section better fits the planned dose distribution, so this should be accounted when a two‐dimensional evaluation is needed. Moreover, the major reliability of the 3D metric with respect to the 2D one, as it better agrees with the dosimetric evaluation on the whole volume, suggests that it should be preferred in a two‐dimensional evaluation. Better agreements, obtained with RA vs IMRT technique, confirm that Octavius^®^‐4D is specifically conceived for rotational delivery. Lastly, the assembly resulted sensitive to different technology.

## 1 INTRODUCTION

A careful pretreatment check with a patient‐related quality assurance protocol (QA) is a crucial step in the pretreatment process in radiation therapy, because of plan complexity and high sophisticated technology behind the radiation delivery. Several tools are available for a two dimensional evaluation, as well as gafchromic films, Electronic Portal Imager Device (EPID), arrays of diodes, (as MapCHECK, SunNuclear Corporation) or of ionization chambers, (as MatriXX, IBA Dosimetry, and 2D‐Array Seven 29™, PTW). Most of them allow to sum multiple irradiations into a single dose plane, applying correction factors in order to take into account the directional dependence on the gantry angle.^1^ However, because of the complex, highly conformal three‐dimensional shape of treatment volumes, a full 3D dose matrix, with a volumetric evaluation of composite fields, is strongly preferable to a planar dose value map.^2^, ^3^, ^4^

In conventional linear accelerators (LINACs), dosimetric distribution corresponding to the different positions of gantry can be obtained by portal imaging and specific software, by algorithms computing the inner dose distribution by interpolation of measurements, as EpiQA^™^ (EPIdos), through the GLAaS algorithm. Other assemblies furnish dosimetric data corresponding to the gantry position using phantoms characterized by opportune geometries or rotation capability, as SunNuclear ArcCHECK^®^, Scandidos Delta4^®^ phantom, the IBA Compass, and PTW Octavius^®^‐4D. Many studies report a comparison of these devices, assessing their different responses as well as their ability in detecting intentional errors,^4^, ^5^, ^6^ or they were used in the validation process of novel QA strategies.^7^, ^8^ The present study is focused on PTW Octavius^®^‐4D. Its main characteristic is that it furnishes a time‐resolved dosimetric acquisition as it rotates together with gantry, and allows the reconstruction of a volumetric dose distribution. The *γ*‐metric, the standard technique used to evaluate the agreement between planned and measured dose,^9^ can be obtained not only in a two‐dimensional way but also three‐dimensionally, allowing the evaluation of the whole volumetric dose distribution. Nowadays, PTW Octavius^®^‐4D is widely used in the QA process, and its performances were already evaluated also for stereotactic treatments,^10^, ^11^, ^12^ with flattening filter free (FFF) technology^13^ or for testing respiratory‐gated VMAT delivery.^14^ In spite of this, some aspects of its performances were only partially investigated.^1^, ^15^, ^16^ This study aims to characterize the 2D‐Array in Octavius^®^‐4D for both IMRT and VMAT delivery techniques. The relationship among the different kinds of *γ*‐index metrics was investigated. The dependence on the phantom axis was assessed as well as the impact of different LINAC and the delivery modality (if IMRT or VMAT). Three different anatomic districts were considered [head and neck (H&N), pelvis, and pancreas] in order to evaluate device response to dose distribution with different modulations and heterogeneities.

## 2 MATERIALS AND METHODS

### 2.A Octavius^®^ 4D: general description

The 2D‐Array together with Octavius^®^‐4D are widely described in the literature.^1^, ^16^ Briefly, the 2D‐Array consisted in a matrix of 729 vented ionization chambers distant 10 mm from center to center, embedded in a 27 × 27 array. Each chamber has the cubic size of 5 × 5 × 5 mm^3^ and the effective measuring point is 7.5 mm below the surface of the detector array. The array is inserted into a motorized cylindrical polystyrene phantom, (diameter and length 32 cm and 34.3 cm respectively). Its capability to rotate synchronously with the gantry, in terms of angle and rotation speed as in the real planned treatment, is made possible, thanks to an inclinometer that is set on the gantry and that is connected to a control unit that transfers the movement information to the phantom and acquires dosimetric data every 200 ms. The beam always hits the detector array in a perpendicular way because the same face of the detector follows the gantry, so no correction factors are required.

The detectors of the 2D‐Array have a calibration certificate for the central chamber in absorbed dose to water, that is independent from the Octavius‐4D phantom (German National Laboratory, PTB, Braunshweing). For all the other chambers of the 2D‐Array, a calibration file provides correction factors respect to the central chamber. So it is possible to perform an intercalibration of the central chamber measuring the dose in the phantom with an ionization chamber inserted into a chamber plate, following the IAEA Technical Report Series 398 approach, and defining a correction factor (*K*_user_). Otherwise, it is possible to obtain the cross‐calibration using, as reference value, the expected dose provided by the TPS in the condition defined by vendors (corresponding to: field 10 × 10 cm^2^, 200 MU, DR 300 MU/min, gantry at 0°) and using the so called *K*_cross_, that is the ratio between the TPS value and the measurement of the central chamber of the array. This permits to take into account the output variations of the LINAC. The obtained correction factor, got by one of the two described methods, is then applied to the whole detector matrix. Dose values are reconstructed first by the conversion of the PDD measured in water for different field sizes to the ones in Octavius^®^‐4D by the relation between the electron densities of water and the phantom material. With this approach the dosimetric information is independent of the TPS. Data are processed by the software package PTW VeriSoft (version 6.1) that elaborates a three‐dimensional grid of dosimetric data. It allows dose evaluation with different metrics, by both local and global *γ*‐index in 2D, 3D for the three axes (axial, sagittal, and coronal) and on the whole volume (volumetric).

### 2.B Octavius^®^‐4D commissioning

The commissioning of Octavius^®^‐4D was carried out with the Trilogy Varian linear accelerator. PDD were measured for field sizes ranging from 4 × 4 cm^2^ to 26 × 26 cm^2^ measured at 85 cm Source to Surface Distance (SSD). The Octavius^®^‐4D CT used for plan verification was the artificial one provided by vendor. However, in order to better model the phantom in the TPS, a CT scan was performed and the obtained averaged HU was reported into the TPS. Moreover, the correct distance from the couch was measured and reported in the artificial CT by fusion of images. Static delivery tests included: 5 × 5, 10 × 10, 5 × 20 cm^2^ static fields, a “pyramid” field‐in‐field shape given by superimposition of 5 × 5, 10 × 10, 20 × 20 cm^2^ static fields. The evaluation of arc delivery performance was obtained using a 5 × 5 cm^2^ arc and the combination of 5 × 5 and 10 × 10 cm^2^ arcs, with a clockwise 180° rotation for both arcs, which were sequentially delivered in order to cover a full rotation of the phantom. The effect of different spatial directions was assessed by considering transversal, coronal, and sagittal planes and verifying whether there was a different response or not. Dosimetric data were assessed by the *γ*‐analysis with acceptance criteria 3%/3 mm.^1^, ^4^

In order to achieve absolute dose, the central chamber of the array was cross‐calibrated with a PTW Semiflex ionization chamber (volume 0.125 cc, type 31010), inserted into a RW3 slab replacing the 2D‐Array inside Octavius^®^‐4D. The position of the chamber with respect to the isocenter was preliminarily verified by orthogonal kV images obtained by mean of the Varian On‐Board Imager (OBI). Measurements at the reference condition were carried out (field 10 × 10 cm^2^, gantry at 0°, 200 MU corresponding to 1.248 Gy for Trilogy and 1.344 Gy for Clinac 2100 DHX, i.e. the expected value in each measure session for the clinical plan verification) and the dose value was deduced from the chamber signal following the IAEA Technical Report Series 398 approach, taking into account also the correction for the daily LINAC output factor. The comparison with the central chamber measurement at the same conditions allowed deducing the so‐called *K*_user_ factor for each LINAC, useful for a validation of Octavius^®^‐4D for absolute dose assessment. This result was compared with the *K*_cross_, in order to evaluate the consistency of the two approaches (see Section 2.A).

Finally, it was evaluated that the dose distribution obtained by two uninterrupted clinical arc deliveries vs the same arcs delivered with up to four interruptions in order to understand the performance of the LINAC when an undesirable arc interruption happens and to test the inclinometer reliability.

### 2.C Treatment plans

The treatment QA delivery was performed by two Varian linear accelerators (Varian Medical Systems, Palo Alto, CA), one the DHX, the other the Trilogy (the only with RapidArc technology), both equipped with 120 leaf Millennium dMLC. The radiation energy was 6 MV for all fields. Dose distributions were computed by the Analytic Anisotropic Algorithm TPS (AAA; Varian Eclipse v. 13.0.26) for both the LINACs. Treatment plans of 44 real clinical cases were evaluated. They were 29 sliding windows IMRT (14 delivered on DHX and 15 on Trilogy, 65.9%) and 15 VMAT RapidArc treatments (only on Trilogy, 34.1%). Treatment regions were pelvis (*n* = 19, 43.2%), head/neck (*n* = 15, 34.1%) and pancreas (*n* = 10, 22.7%). A summary of treatment plan characteristics is reported in Table 1, while each treatment plan is detailed in Supporting Information — Table S1.

**Table 1:** Description of main treatment plan characteristics, grouped for studied anatomical region

Treatment plan description										
Site	*n*	LINAC		Technique		Plan characteristics				
		DHX	Trilogy	IMRT	RA	SIB	Gy/fx	Target volume (cc)	N fields/arcs	Jaw setting (cm)
H&N	15	5	10	10	5	15	1.7/1.9/2	175.8/60.2/87.5	5‐7‐8/2‐3	17.2 × 16.6
Pelvis	19	9	10	14	5	9	1.9/2.1/2.3	252.4/99.2/43.7	5‐6‐7‐9/2	18.1 × 16.9
Pancreas	10	–	10	5	5	4	1.9/2.0	293.7/102.9	5‐7‐8/2	14.5 × 12.8

The dosimetric verification was carried out comparing the measured plan with the so‐called “verification plan”, where the dose distribution of the treatment plan was recalculated on the CT of the phantom performed with the in‐home CT scan (the same used for commissioning), with a slice thickness of 3 mm. The TPS grid was set at 2.5 mm.

### 2.D Evaluation of dose distribution

2D, 3D and the volumetric *γ* were evaluated with 3%/3 mm acceptance criterion,^4^ because of its prevalent use in clinical practice,^17^ and DD in local *γ* analysis was increased to 5% for doses lower than 0.1 Gy. Results by both local and global *γ* analysis definitions were investigated. The Γ < 1 was required to be satisfied at least in 95% of points. The analysis of results has been carried out by no ROI selection, as the whole volume was considered with a cut‐off threshold set to 5% with respect to the maximum dose. This choice was coherent with threshold reported by an American survey of Nelms and Simon^18^ (as more than 70% of 139 institutions involved in the study used a threshold between 0% and 10%).

### 2.E Statistical analysis

Statistical analysis was carried out by the software package SPSS.20 (SPSS, Inc., Chicago, IL; USA). The gaussian distribution of samples was tested by Kolmogoroff–Smirmoff test after log_e_‐transformation to reduce heteroscedasticity. Analysis of variance was studied for comparison of different groups and *t*‐paired test for paired data (respectively, Kruskal–Wallis test for independent groups or Wilcoxon test for paired data if not parametric test was required). The significance level was set at *P* < 0.05.

## 3 RESULTS

### 3.A Octavius^®^‐4D commissioning

**Figure 1:**
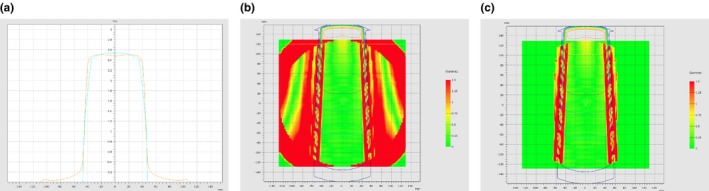
(a–c) Comparison between measured and calculated dose distribution in a 10 × 10 cm static field (gantry set at 0°) in the Trilogy LINAC by VeriSoft with Octavius‐4D. (a) Dose profile comparison. (b) Local gamma analysis 3%/3 mm with no cut‐off for low threshold analysed by VeriSoft (boundary layer of the phantom are visible). (c) Local gamma analysis 3%/3 mm with 5% cut‐off of maximum dose analysed by VeriSoft.

For the Octavius^®^‐4D preliminary commissioning, dose profile for static 10 × 10 cm is reported in Fig. 1(a). The low‐dose threshold was initially set at 0, but this revealed that the gamma index computation by the VeriSoft algorithm took into account also the boundary layer of the phantom, where no detectors were present [see Fig. 1(b)]. It was found that a 0.1% of the maximum dose was a sufficient low‐dose threshold for the exclusion of this inconsistent area of comparison. Then the cut‐off threshold was set at 5% of maximum dose, obtaining the change in agreement showed in Fig. 1(c), where the other delivery characteristics are unmodified. The picture shows that the boundary layer appeared in good agreement, as it was not included in comparison, while the gradient area remained with scarce agreement.

**Figure 2:**
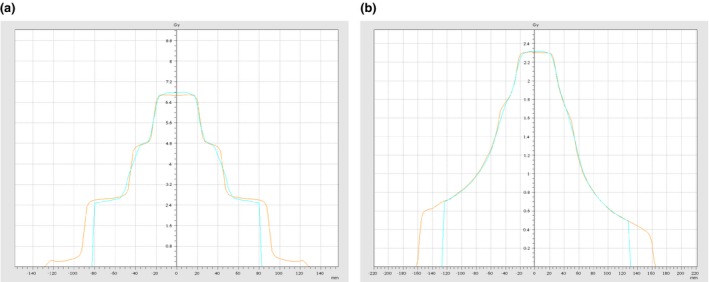
(a–b) Dose profile comparison between measured and calculated dose in the Trilogy LINAC analysed by VeriSoft with Octavius‐4D during commissioning. The static pyramid test (a) and the double arc test (b) are reported.

The accordance among measured and calculated fields gave a percentage of passing points with global volumetric *γ*‐index ranging from 95% to 100% for both delivered static and rotational fields. However, results obtained with local *γ*‐index fault down to an average value of 51.0%. The discrepancies were evident at the boundaries of the static fields, where the dose falls from 2 Gy to zero [Fig. 1(c)]. In RA delivery, local volumetric *γ*‐index increased up to 62.9%. Dose profiles for static “pyramid” field‐in‐field and double arc tests are reported in Figs. 2(a) and 2(b), respectively, showing worse agreement in the regions of dose variation.

The comparison of agreement for the three different axes showed a different behavior, as the transversal one gave the best agreement (∆ = 15.6% and 27.1% for sagittal and coronal respectively).

The test on the arc interruptions indicated a correct angle recovery and dose delivery after the interruptions. The interrupted arcs were completed obtaining the delivered doses exactly corresponding to those of the uninterrupted arcs (local *γ*‐index 3%/3 mm = 99.9% on the average).

Finally, the measurements of absolute dose performed by mean of the ionization chamber showed a 5.4% difference for DHX and 4.7% for Trilogy with respect to the dose measured by the central chamber of the PTW 2D Array (inserted in Octavius^®^‐4D), that is provided by the VeriSoft software itself during the acquisition of the dose measurement. These values, that represented the *K*_user_ indicated in the VeriSoft software, were in accordance with the difference between the TPS value and the measurement of the central chamber in the PTW 2D Array (on the average 5.2% for the DHX and 5.7% for the Trilogy). As the difference between *K*_user_ and *K*_cross_ was ≤1% for both the LINACs, this results confirmed the consistency of the two approaches.

### 3.B Dose distribution verification

Pretreatment plan verification by mean of Octavius^®^‐4D resulted in a percentage of at least 90% passed points for 75.0% of cases for local volumetric *γ*‐index (average value = 91.5 ± 4.1%) that rose up to 100% for global volumetric *γ*‐index (average value = 97.9 ± 1.8%).

#### 3.B.1 Dependence on the *γ*‐metric

The choice of the *γ*‐metric implied different results in plan evaluation. The results evaluated on the three axes were resumed in their mean value in order to take into account the three dimensions together.

**Figure 3:**
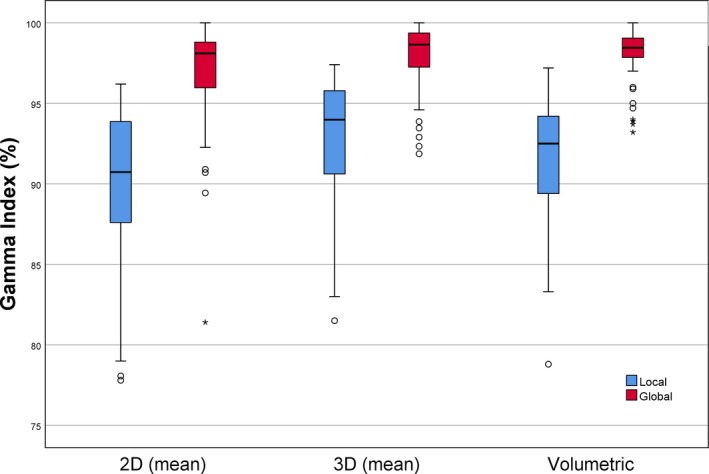
Comparison among the volumetric local and global *γ*‐index and the corresponding 2D and 3D *γ*‐index averaged on the three axes (transversal, sagittal, and coronal) obtained by measurements with Octavius^®^‐4D.

The distribution of the volumetric local and global *γ*‐index and the corresponding 2D and 3D *γ*‐index averaged on the three axes (transversal, sagittal, and coronal) are showed in Fig. 3, and their averages in Table 2.

**Table 2:** Arithmetic mean (AM) and standard deviation (SD) of 2D, 3D and volumetric *γ*‐index of the sample. Both local and global *γ*‐metrics are reported. ^*^Average on the 3 axes

*γ*‐index (%) (AM ± SD)			
*n* = 44			
	2D*	3D*	Volumetric
Local	89.7 ± 5.0	92.6 ± 4.1	91.5 ± 4.1
Global	96.7 ± 3.5	97.8 ± 2.3	97.9 ± 1.8

As completely expected, the *γ*‐index calculated with respect to the maximum point (i.e. global) was significantly higher than the one calculated on a point‐by‐point basis (i.e. local; *P* < 0.001).

The comparison between the average values of the *γ*‐index evaluated on the three axes in 2D, 3D metric and the volumetric *γ*‐index showed comparable results between the 3D and volumetric *γ*‐index for the global metric (*P* = 0.343). The 2D *γ*‐index showed statistically significant lower values with respect to the other metrics (*P* < 0.001).

#### 3.B.2 Dependence on the phantom axes

Results of agreement in dosimetric distribution depending on the section of the phantom, if transversal, sagittal, or coronal, were evaluated and results are reported in Table 3.

**Table 3:** Arithmetic mean (AM) and standard deviation (SD) of 2D, 3D *γ*‐index referred to the different axes of the phantom Octavius^®^‐4D

	*n* = 44	*γ*‐index (%) (AM ± SD)		
		Transversal	Sagittal	Coronal
2D	Local	93.4 ± 5.6	86.7 ± 6.5	88.9 ± 4.9
	Global	96.1 ± 0.9	95.3 ± 1.0	93.9 ± 1.2
3D	Local	95.7 ± 5.2	89.3 ± 6.0	92.7 ± 3.1
	Global	98.5 ± 2.5	97.2 ± 3.4	97.8 ± 1.6

The ANOVA test gave a statistically significant difference among the three sections for local *γ*‐index, both 2D and 3D (*P* < 0.001). The Bonferroni test showed that the difference was due to the higher value in the transversal direction with respect to the others (*P* ≤ 0.001). For the global *γ*‐index, the difference was less significant (*P* = 0.034 and *P* = 0.063 for 2D and 3D respectively). *γ*‐statistic of the three sections was always correlated with the volumetric *γ*‐index (*R* ≥ 0.9, *P* < 0.001). If global *γ*‐index was considered, the *γ*‐index evaluated in the only transversal section was statistically comparable with the 2D volumetric one (*P* = 0.192), while in the local approach no associations were found.

#### 3.B.3 Dependence on LINAC and algorithm

The difference in dose delivery between DHX and Trilogy was evaluated. In order to improve homogeneity, the comparison was performed between only IMRT plans, delivered on both LINACs. Average values of *γ*‐indexes are reported in Table 4(a). The differences between the two LINACs performances were statistically significant for both local and global volumetric *γ*‐metric (*P* < 0.001 and *P* = 0.003 respectively).

**Table 4:** (a–b) Arithmetic mean (AM) and standard deviation (SD) of volumetric local and global *γ*‐index of the sample by measurements acquired with Octavius^®^‐4D (a) for the two LINACs for the only IMRT plans, (b) for the only Trilogy LINAC delivered with both IMRT and RA techniques

Volumetric *γ*‐index (%) (AM ± SD)				
	(a)		(b)	
	DHX (*n* = 14)	Trilogy (*n* = 15)	IMRT (*n* = 15)	RA (*n* = 15)
Local	87.2 ± 3.4	93.1 ± 2.1	93.1 ± 2.1	94.0 ± 2.1
Global	96.2 ± 2.1	96.2 ± 2.1	98.6 ± 0.5	99.1 ± 0.6

#### 3.B.4 Dependence on the delivery technique

It was investigated whether the different techniques (IMRT and RA) used for treatment plans lead to difference in passing rates for measurements with Octavius^®^‐4D or not. The average values for the only Trilogy LINAC are reported in Table 4(b). Results showed that *γ*‐index of IMRT is always lower than *γ*‐index for RA technique, even if the difference was statistically significant for only the global metric (*P* = 0.019).

#### 3.B.5 Dependence on the treatment region

The average values of *γ*‐index for the three studied districts are summarized in Table 5. Even if the *γ*‐index average values resulted always lower for head and neck treatments, the differences showed a not significant passing rates trend (*P* = 0.541 and *P* = 0.100 for local and global *γ*‐index). This result is confirmed when considering separately the IMRT and RA techniques at the Trilogy (respectively, *P* = 0.709 and *P* = 0.573 for volumetric local *γ*‐index and *P* = 0.573 and *P* = 0.065 for volumetric global *γ*‐index). Also if selecting the only IMRT plans for both the LINACs, the difference among anatomical regions was never significant (*P* = 0.141 and *P* = 0.488 for local and global *γ*‐index).

**Table 5:** Arithmetic mean (AM) and standard deviation (SD) of volumetric local and global *γ*‐index of the sample by measurements acquired with Octavius^®^‐4D for the three studied anatomic regions (H&N, pelvis and pancreas) delivered on the DHX with only IMRT and on the Trilogy with both IMRT and RA techniques

	Volumetric *γ*‐index (%) (AM ± SD)										
	H&N				Pelvis				Pancreas		
	Total (*n* = 15)	DHX	Trilogy		Total (*n* = 19)	DHX	Trilogy		Total (*n* = 10)	Trilogy	
		IMRT (*n* = 5)	IMRT (*n* = 5)	RA (*n* = 5)		IMRT (*n* = 9)	IMRT (*n* = 5)	RA (*n* = 5)		IMRT (*n* = 5)	RA (*n* = 5)
Local	90.6 ± 4.3	85.5 ± 1.9	92.5 ± 2.7	93.7 ± 1.9	91.3 ± 4.5	88.2 ± 4.5	93.1 ± 1.5	95.3 ± 1.8	92.8 ± 2.2	93.7 ± 2.3	92.8 ± 2.2
Global	97.5 ± 2.2	94.7 ± 1.5	98.6 ± 0.5	99.1 ± 0.6	98.1 ± 1.8	97.0 ± 2.1	98.7 ± 0.6	99.6 ± 0.5	98.8 ± 0.4	98.3 ± 0.5	98.8 ± 0.4

## 4 DISCUSSION AND CONCLUSION

Results underline that several factors affect plan evaluation when using Octavius^®^‐4D, and they are especially enhanced if the more restrictive local *γ*‐index computation approach is used. Indeed, the global *γ*‐index produces more homogeneous results with higher passing rates (Fig. 3), because its tolerance level is computed with respect to the value of maximum dose. The first important issue is related to how the phantom is conceived, as it allows to consider different typology of metrics for the *γ*‐index. The 2D approach considers each slice as independent of the surrounding volume, with the drawback that results are strongly dependent on the chosen plane, without a certain significant correlation between the magnitude of errors of different plans.^19^ Such an aspect is then an undesirable characteristic of the 2D *γ*. The 3D analysis allows a slice‐by‐slice evaluation taking into account also the neighboring slices. The “extra” 3rd dimension used to search for agreement leads to a lower 3D *γ*‐index with respect to the 2D, producing higher passing rates for patient QA if the same acceptance criteria are chosen.^20^, ^21^ Finally, the assessment of the entire volume under study, with a volumetric *γ* evaluation, is probably the main strength of this kind of phantom, especially if considering that it is exposed to the radiation in a time‐resolved mode. Our results confirmed that the single slice evaluation (2D) had always a worse agreement compared to 3D and volumetric *γ*‐index (*P* < 0.001; Fig. 3). Pulliam et al.^17^ compared the two gamma results using a Monte Carlo computation as reference dose distribution and quantified the increase of passing pixels percent up to 3.2% in the 3D analysis, confirming our findings. They also reported that a randomly selected plane provided a passing gamma value (2D) consistent with those provided by the majority of other planes but not always with the entire patient treatment volume. Interesting, when considering the 3D *γ*‐index, our results showed that the mean on the three axes is statistically comparable with the volumetric global *γ*‐index (*P* = 0.343; Table 2). On the contrary the 2D evaluation (averaged on the three axes) was not comparable. This finding suggests that the evaluation of a slice with the neighboring ones (3D) can be a good proxy of the agreement between calculated and measured dose distribution on the entire volume, conversely to a 2‐D evaluation, as reported in Rajasekaran et al.^22^

Results also indicated a dependence on the section where the plan was evaluated: indeed the transversal section was always linked to a better agreement if compared with the coronal and the sagittal ones (*P* ≤ 0.001) and it gave global *γ*‐index comparable with the 2D volumetric passing rates (*P* = 0.192; Table 3). As reported by vendors, the transversal view is most easily related to the treatment plan isodose on the transversal CT slice of the patient. Moreover, once the 3D dose reconstruction grid is set as required by the VeriSoft code of practice (voxel side length: *x* = 2.5 mm, *y* = 10 mm, *z* = 2.5 mm), the plane coordinates of the measured and calculated dose matrix have to correspond, while, for the coronal and sagittal views, the control over the exact pixel position is scarcer.

Different agreements were obtained depending on the LINAC used for QA [*P* ≤ 0.003; Table 4(a)]. This dependence can be explained by the use of the different technology, as the stricter tolerances of the components of the Trilogy provide better consistency with the computation model than for the oldest DHX. The interesting point of such a result is that Octavius^®^‐4D is able to detect difference in delivery technology, considering the same technique (only IRMT) and computation algorithm (AAA).

Poor agreement, obtained with static field at the commissioning step, is linked to the overly smoothened dose reconstruction due to the combined effect of the detector resolution and the interpolation performed by the algorithm [Fig. 1(c)]. This behavior was already underlined by Allagaier et al.^1^ and it is linked to the low sampling of measurements, that affects the correctness of the algorithm interpolation. Such a problem is also present in IMRT plans, where the resolution of the detector can be inadequate in steep dose profiles, as the linear interpolation of measurements in a discrete array of detectors can produce an overly smoothed dose profile reconstruction. So the detector resolution together with the algorithm lead to a poor agreement when the dose profile is more modulated.^15^, ^23^, ^24^ On the contrary, in RapidArc delivery, the larger amount of acquired data produces a better estimation of measured dose. The rotation of the array together with the gantry and the acquisition of data in different position during the rotation allow a better estimate of the dose distribution by the software. This leads to the significant improvement of agreements with respect to the IMRT technique, as confirmed by results in Table 4(b) (*P* = 0.019).

No dependence on the treated regions was found, as the difference in *γ*‐index was not statistically significant (*P* > 0.065; Table 5). This result indicated that the VeriSoft algorithm similarly manages the different typical dose distribution for the three studied districts in Octavius^®^‐4D. However, even if the difference was not significant, the *γ*‐index was lower for H&N. This result was not surprising, as this kind of treatments generally require very high modulation in small target volumes. Then, the resolution of the Array‐729 used in Octavius‐4D (where the ionization chambers are distant 1 cm from center‐to‐center) can be more critical than for other anatomical districts.

Finally, the comparison between the absolute dose and the dose measured by the central chamber of the array, assessed during the commissioning of the phantom for the *K*_user_ determination, furnished worse agreement than what was reported in the literature (about 5% difference for both the LINACs vs 1–2% in Stathakis et al.)^16^, probably due to array‐related characteristics. However, it was found that the *K*_user_ was comparable with the *K*_cross_, obtained by the comparison with the TPS expected value (Δ ≤ 1%). The consistency of the two approaches suggests that the cross‐calibration, necessary to take into account the daily output variation of the LINAC, could be performed by using the *K*_cross_ approach, which is simpler than the *K*_user_ approach.

In conclusion, the study pointed out that Octavius^®^‐4D is a very reliable tool, especially for VMAT pretreatment quality assurance, as very good agreements of treatment plans delivered with RA technique were found. The system resulted sensitive enough to catch differences in LINAC technology while it similarly managed pancreas, pelvis, and H&N treatments. A useful finding was that, in a two‐dimensional evaluation, the study showed that the transversal section better fits the planned dose distribution. Moreover, 3D slice evaluation is generally comparable with volumetric evaluation, suggesting that it should be preferred to the 2D when volumetric metric is not available.

## CONFLICT OF INTEREST

None declared.

## Supporting information


**Table S1**. Single plan details.
