# Supplementary material for: Practical application of Octavius®‐4D: Characteristics and criticalities for IMRT and VMAT verification
Source: J Appl Clin Med Phys. 2018 Jul 16;19(5):517–24. doi: 10.1002/acm2.12412 (PMC6123102; doi:10.1002/acm2.12412)
Supplement: Supplementary file 1 — Table S1. Single plan details. [file ACM2-19-517-s001.docx]

| **Patient ID** | **LINAC** | **Anatomic region** | **Technique IMRT/RA** | **Gy/fx** | **N**  **arcs** | **N fields** | **Jaw settings**  **[cm]** | **Target volume**  **[cc]** |
| --- | --- | --- | --- | --- | --- | --- | --- | --- |
| 1 | DHX | H&N | IMRT | 1.67 - 2 |  | 7 | 17.3 x 18 | 154/31.7 |
| 2 | DHX | H&N | IMRT | 1.6 -1.8 -2 |  | 7 | 21.6 x 9.8 | 363.3/248.7/226.7 |
| 3 | DHX | H&N | IMRT | 1.65 -1.9 - 2 |  | 7 | 25.3 x 20.1 | 401.5/139.4/40.8 |
| 4 | DHX | H&N | IMRT | 1.7 -2 |  | 5 | 13.3 x 18.5 | 229.2/63 |
| 5 | DHX | H&N | IMRT | 1.8 -2 |  | 7 | 11.5 x 12.5 | 176.5/67.3 |
| 6 | TRILOGY | H&N | IMRT | 1.8 - 2.14 |  | 7 | 20.6 x 15.3 | 134.5/31.6 |
| 7 | TRILOGY | H&N | IMRT | 1.6 - 1.8 - 2 |  | 7 | 17.5 x 19.8 | 124.1/7.1/50 |
| 8 | TRILOGY | H&N | IMRT | 1.8 - 2 |  | 7 | 17 x 12.6 | 97.2/22.3 |
| 9 | TRILOGY | H&N | IMRT | 1.66 - 1.8 - 2 |  | 8 | 20.6 x 22.8 | 203.4/60.5/108 |
| 10 | TRILOGY | H&N | IMRT | 1.66 - 1.8 - 2 |  | 8 | 17.4 x 18.3 | 119.2/41.8/91.9 |
| 11 | TRILOGY | H&N | RA | 1.8 - 2.1 | 2 |  | 13.1 x 17.3 | 43.2/32.1 |
| 12 | TRILOGY | H&N | RA | 1.66 - 2 | 2 |  | 11.1 x 17.3 | 158.3/32.2 |
| 13 | TRILOGY | H&N | RA | 1.62 - 1.8 - 2 | 2 |  | 18 x 13.3 | 107/3.8/100 |
| 14 | TRILOGY | H&N | RA | 1.7 - 1.8 - 2 | 2 |  | 17.2 x 18.7 | 102.5/61.2/29.3 |
| 15 | TRILOGY | H&N | RA | 1.66 - 1.8 - 2 | 3 |  | 16 x 22.8 | 222.8/90.2/53.6 |
| 16 | DHX | Pelvis | IMRT | 1.8 |  | 7 | 24.5 x 24.3 | 1081.4 |
| 17 | DHX | Pelvis | IMRT | 2 |  | 7 | 20.8 x 17 | 257.6 |
| 18 | DHX | Pelvis | IMRT | 1.8 |  | 7 | 20.5 x 21.8 | 312.7 |
| 19 | DHX | Pelvis | IMRT | 1.8 |  | 7 | 21.9 x 20 | 233.6 |
| 20 | DHX | Pelvis | IMRT | 1.8 |  | 7 | 21.6 x 21.6 | 388.5 |
| 21 | DHX | Pelvis | IMRT | 1.8 - 2.3 |  | 6 | 10.8 x 9.8 | 72/65 |
| 22 | DHX | Pelvis | IMRT | 2.2 - 1.6 |  | 9 | 13x 8.8 | 27.3/68.6 |
| 23 | DHX | Pelvis | IMRT | 2 |  | 5 | 12.7 x 5.8 | 37.8 |
| 24 | DHX | Pelvis | IMRT | 2 |  | 7 | 21.9 x 12.3 | 431.7 |
| 25 | TRILOGY | Pelvis | IMRT | 1.85 - 2.1 - 2.3 |  | 7 | 19.9 x 19.1 | 334.5/31.1/65.1 |
| 26 | TRILOGY | Pelvis | IMRT | 1.8 |  | 7 | 20.1 x 23.6 | 377.2 |
| 27 | TRILOGY | Pelvis | IMRT | 1.8 - 2.3 |  | 7 | 21.3 x 16.5 | 144.6/49.4 |
| 28 | TRILOGY | Pelvis | IMRT | 1.8 - 2 |  | 7 | 14.8 x 24.3 | 32.4/564.9 |
| 29 | TRILOGY | Pelvis | IMRT | 1.75 - 2.1 - 2.3 |  | 6 | 20.8 x 21.3 | 281.3/10.6/43.7 |
| 30 | TRILOGY | Pelvis | RA | 1.96 - 2.21 | 2 |  | 13 x 11.8 | 57/35 |
| 31 | TRILOGY | Pelvis | RA | 1.8 | 2 |  | 20 x 20.8 | 400.3 |
| 32 | TRILOGY | Pelvis | RA | 1.9 - 2.1 | 2 |  | 12.8 x 9.8 | 7/26 |
| 33 | TRILOGY | Pelvis | RA | 2.35 - 2.1 | 2 |  | 14.1 x 11.6 | 42/42 |
| 34 | TRILOGY | Pelvis | RA | 1.8 | 2 |  | 20 x 20 | 305.2 |
| 35 | TRILOGY | Pancreas | IMRT | 1.8 - 1.95 |  | 8 | 15.1 x 9.8 | 368.1/47.7 |
| 36 | TRILOGY | Pancreas | IMRT | 1.8 - 2.0 |  | 7 | 13.2 x 15 | 323.2/137.3 |
| 37 | TRILOGY | Pancreas | IMRT | 2 |  | 5 | 14.1 x 12.1 | 239,9 |
| 38 | TRILOGY | Pancreas | IMRT | 2 |  | 8 | 13.5 x 16 | 349,1 |
| 39 | TRILOGY | Pancreas | IMRT | 1.8 |  | 5 | 14.8 x 11.3 | 296,6 |
| 40 | TRILOGY | Pancreas | RA | 1.8 | 2 |  | 14 x 10 | 114,8 |
| 41 | TRILOGY | Pancreas | RA | 2 | 2 |  | 17.2 x 13.9 | 438,3 |
| 42 | TRILOGY | Pancreas | RA | 1.8 - 2.1 | 2 |  | 16.5 x 16 | 381.3/118.4 |
| 43 | TRILOGY | Pancreas | RA | 1.8 | 2 |  | 11.8 x 10.5 | 124,2 |
| 44 | TRILOGY | Pancreas | RA | 18 - 2.1 |  |  | 14.9 x 13.2 | 301.4/108.2 |

SM - Table 1 Single plan details
